# The Biflavonoid Agathisflavone Regulates Microglial and Astrocytic Inflammatory Profiles via Glucocorticoid Receptor

**DOI:** 10.3390/molecules30051014

**Published:** 2025-02-22

**Authors:** Áurea Maria Alves Nunes Almeida, Cleonice Creusa dos Santos, Daniele Takahashi, Larissa Pereira da Silva, Verônica Moreira de Sousa, Monique Reis de Santana, Ana Elisa Del Arco, Balbino Lino dos Santos, Jorge Mauricio David, Victor Diogenes Amaral da Silva, Suzana Braga-de-Souza, Silvia Lima Costa

**Affiliations:** 1 Laboratory of Neurochemistry and Cellular Biology, Institute of Health Sciences, Federal University of Bahia, Av. Reitor Miguel Calmon S/N, Salvador 40231-300, Brazil; aurea.maria.almeida@gmail.com (Á.M.A.N.A.); cleonicemev@gmail.com (C.C.d.S.); daniele.takahashi@ufba.br (D.T.); lariipedreira@hotmail.com (L.P.d.S.); veumoreirasousa@gmail.com (V.M.d.S.); moniquereisant@gmail.com (M.R.d.S.); anaelisa@ufrb.edu.br (A.E.D.A.); balbino.lino@univasf.edu.br (B.L.d.S.); vdsilva@ufba.br (V.D.A.d.S.); 2 Laboratory of Biochemistry and Veterinary Immunology, Center for Agrarian, Environmental, and Biological Sciences, Federal University of Recôncavo of Bahia, Cruz das Almas 44380-000, Brazil; 3 College of Nursing, Federal University of Vale do São Francisco, Petrolina 56304-917, Brazil; 4 Department of General and Inorganic Chemistry, Institute of Chemistry, University Federal da Bahia, Salvador 40170-110, Brazil; jmdavid@ufba.br; 5 National Institute of Translational Neuroscience (INNT), Rio de Janeiro 21941-902, Brazil

**Keywords:** anti-neuroinflammatory, IL-10, astrocyte, microglia, neuroprotection, flavonoid, glucocorticoid receptor

## Abstract

Nuclear receptors such as glucocorticoid receptors (GRs) are transcription factors with prominent regulatory effects on neuroinflammation. Agathisflavone is a biflavonoid that demonstrates neurogenic, neuroprotective, anti-inflammatory, antioxidant, and pro-myelinogenic effects in vitro. This study investigated whether the control of glial reactivity by agathisflavone is mediated by GRs. Primary cultures of astrocytes and microglia were induced to neuroinflammation by lipopolysaccharides (LPSs) and exposed to agathisflavone or not in the presence or absence of mifepristone, a GR antagonist. The microglia morphology and reactivity were evaluated by immunofluorescence against calcium-binding ionized adapter (Iba-1) and CD68. The astrocyte morphology and reactivity were evaluated by immunofluorescence against glial fibrillary acidic protein (GFAP). The inflammatory profile was evaluated by RT-qPCR. Molecular docking was performed to characterize agathisflavone and GR interactions. Microglial branching was increased in response to agathisflavone, an effect that was inhibited by mifepristone. CD68 and GFAP expression was decreased by agathisflavone but not in the presence of mifepristone. Agathisflavone decreased the expression of the pro-inflammatory cytokine IL-1β and increased the expression of the regulatory cytokine IL-10. The increase in IL-10 mRNA was inhibited by the GR antagonist. The in silico analysis showed that agathisflavone binds to a pocket at the glucocorticoid receptor. These interactions were stronger than mifepristone, dexamethasone, and the agathisflavone monomer apigenin. These results indicate that the GR is involved in the regulatory effects of agathisflavone on microglia and astrocyte inflammation, contributing to the elucidation of the molecular mechanisms of agathisflavone’s effects in the nervous system.

## 1. Introduction

Neurodegeneration is the progressive neuronal death that results in cognitive and motor deficits during aging, with subsequent morbidity and mortality [1]. Neuroinflammation is a common feature underlying neurodegenerative diseases with different etiologies. It is an acute or chronic defense response of the organism to signs of damage that trigger an inflammatory cascade involving cross-interactions between glial cells and neurons. These events, followed by changes in gene expression and cytokine release, affect neuronal receptors and promote glial activation [2].

Several cell types contribute to inflammation in the central nervous system (CNS), releasing pro-inflammatory cytokines in response to damage. Microglia act as resident macrophages of the CNS and are the first line of defense against inflammatory stimuli [3,4], and when activated, they acquire morphological and molecular characteristics comprising multifaceted responses, depending on the region of the tissue and the nature of the stimulus that generated the activation. In the pro-inflammatory state, microglia release pro-inflammatory, apoptotic, or neurotoxic factors, while in the regulatory state, they release regulatory and neurotrophic factors [5]. Astrocytes also exhibit a diversity of activation states in response to pro-inflammatory stimuli mediated by microglia [6]. The neurotrophic and regulatory activation profiles of microglia and astrocytes are associated with the resolution of the inflammatory response, and the mechanisms that induce these profiles have been investigated as potential therapeutic targets against chronic neuroinflammation [7,8].

Flavonoids are polyphenolic compounds that are products of the secondary metabolism of plants with several demonstrated effects (e.g., neuroprotective, anti-inflammatory, antioxidant, neurogenic, antimicrobial, antitumor) demonstrated in different experimental contexts. Due to their therapeutic potential, the molecular mechanisms involved in the effects of flavonoids have also been investigated in the context of neurodegenerative diseases [9]. The biflavonoid agathisflavone is a dimer of apigenin found in several plant families [10]. Previous studies have shown that agathisflavone has a pro-neurogenic action capable of enhancing neurogenesis via the retinoic acid receptor (RAR) [11]. Another study from our group demonstrated that agathisflavone has a neuroprotective effect against glutamate-induced excitotoxicity via estrogen receptors [12]. More recently, the anti-neuroinflammatory, pro-myelinogenic, and antioxidant effects of agathisflavone were also demonstrated in vitro, effects mainly associated with the modulation of the microglia inflammatory profile [12,13].

The glucocorticoid receptor (GR) is a protein of the nuclear receptor superfamily that, in response to a ligand, is translocated to the nucleus and acts as a transcription factor, repressing or inducing gene expression. The nuclear receptor superfamily comprises 48 proteins, including mineralocorticoid, estrogen, progesterone, androgen, and retinoic acid receptors, whose domains share high structural and functional similarities [14]. Due to its plasticity and its expression being widely distributed in the body, the GR can exert multiple effects, depending on the tissue in which it is expressed. However, one of its broadest effects is the regulatory and immunosuppressive action through the inhibition of the synthesis of pro-inflammatory mediators such as nuclear factor-κB (NF-κB) [14]. In microglia in vitro, GR inhibits the synthesis of pro-inflammatory mediators [15] and, in astrocytes, it inhibits proliferation in vitro and reduces GFAP expression in vivo [16].

Considering these data, this study evaluated if the regulatory action of agathisflavone on microglia and astrocytes is mediated by GR in an in vitro model of neuroinflammation. We also analyzed, by molecular docking, the interaction of agathisflavone with GR in comparison with mifepristone (a GR antagonist), dexamethasone (GR agonist), and the flavonoid apigenin, the agathisflavone monomer.

## 2. Results

### 2.1. Effects of Agathisflavone and RU486 on Glial Cell Viability

**Figure 1:**
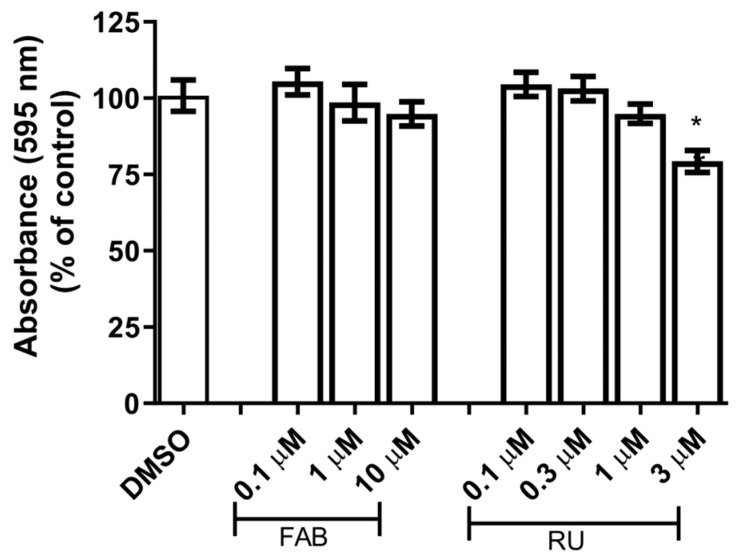
Cytotoxicity of different concentrations of agathisflavone (FAB) and RU486 (RU, a GR antagonist) evaluated by MTT test in glial cell primary cultures. Glial cell cultures were treated with different concentrations of agathisflavone (0.1 µM, 1 µM, and 10 µM) and RU486 (0.3 µM, 1 µM, and 3 µM) for 24 h. Means of absorbance values at 595 nm (*y*-axis) for each experimental group (*x*-axis) are expressed as a percentage of the control (DMSO 0.003%), considered as 100%. The asterisks on the bars indicate a statistical difference relative to control, calculated using the Mann–Whitney test. * *p* < 0.05.

In this study, to evaluate if the regulatory action of agathisflavone on microglia and astrocytes is mediated by GR, we preformed glial (astrocytes/microglia) primary cultures enriched with astrocytes obtained from the cortex of newborn Wistar rats. Primarily, after 15 days in vitro (DIV), the cultures were maintained in control conditions (DMSO 0.003%) or treated with agathisflavone (0.1; 1; and 10 µM) or with mifepristone (RU486) (0.1; 0.3; 1; and 3 µM), a GR antagonist. The viability of glial cells was assessed by the 3-(4,5-dimethylthiazol-2-yl)-2,5-diphenyltetrazolium bromide (MTT) test after 24 h exposure, an assay that measures the activity of mitochondrial dehydrogenases. The choice of the concentrations tested was based on previous studies that demonstrated agathisflavone neurogenic and/or anti-inflammatory effects [12,13]. Agathisflavone had no effect on the viability of glial cells at tested concentrations. However, RU486 at 3 µM induced a significant reduction in cell viability relative to control (Figure 1). Hence, for the next steps of the study, we adopted equimolecular concentration of 1 µM for agathisflavone and RU486.

### 2.2. Effects of Agasthisflavone and the GR Antagonist RU486 on LPS-Induced Microglial Activation

To elicit an inflammatory response, glial cell cultures were treated with *Escherichia coli* lipopolysaccharide (LPS) and treated with agathisflavone alone or in the presence of mifepristone (RU486), and the effects on astrocyte and microglia activation and inflammatory markers were characterized in the following conditions: i. in response to the LPS (1 µg/mL); ii. in response to agathisflavone (1 µM) and LPS together; iii. in response to pretreatment with mifepristone (1 µM) for 2 h and subsequent addition of agathisflavone and LPS. All treatments with agathisflavone and/or LPS were performed for 24 h. Proliferation, changes in the morphology and expression of the surface of pattern recognition receptors, or clusters of differentiation (CDs) are hallmarks of microglial activation and response to external insults, such as the inflammatory response.

**Figure 2:**
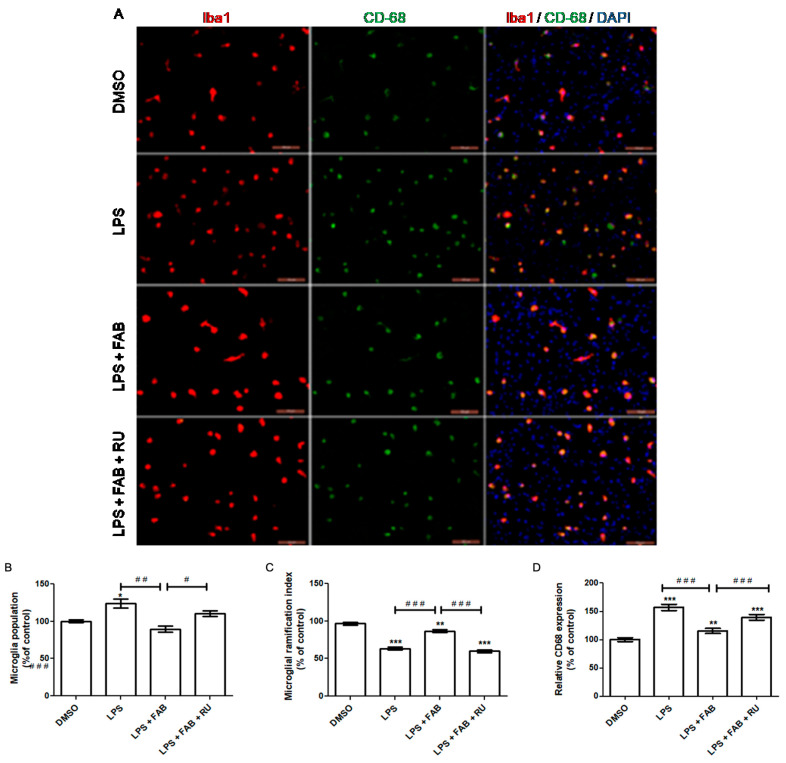
Evaluation of the involvement of GR in agathisflavone effects on LPS-induced microglial reactivity. Glial cells were pretreated or not with the GR antagonist RU486 (RU, 1 µM) for 2 h and treated with LPS (1 µg/mL) alone or in association with agathisflavone (FAB, 1 µM) for 24 h. (**A**) Photomicrographs of immunofluorescent staining for Iba-1 (red) and CD68 (green), counterstained with DAPI (blue) for the nuclei. (**B**) Quantification of microglial population in each experimental group. (**C**) Quantification of microglial ramification in each experimental group. (**D**) Quantification of CD68 expression in each experimental group. The results are expressed as the mean ± standard deviation. The asterisk (*) on the bars indicates a statistical difference relative to control (DMSO 0.001%), which was considered as 100%. The hashes (#) indicate statistical differences between groups; the number of asterisks or hashes correspond to the significance of the statistical difference calculated using the Mann–Whitney test (α = 0.05) according to the *p* value: *** *p* < 0.0001; ** *p* < 0.001, * *p* < 0.01, ### *p* < 0.0001, ## *p* < 0.001, # *p* < 0.01. Scale bars 100 µm.

Microglia cells were identified in the glial cultures by immunofluorescence against the ionized calcium-binding adapter molecule 1 (Iba-1), a specific cytoskeletal marker, and their proportion in the cultures was measured by assessing the percentage of Iba-1+ cells per total cells (Figure 2A–C). Relative to the control, LPS increased the microglial population (*p* < 0.0001); however, this increase was inhibited in the presence of agathisflavone, with the microglial population similar to control cultures. Prior treatment with mifepristone reversed the effect of agathisflavone, and the microglia population was increased (*p* < 0.01).

The microglial morphology was evaluated by the degree of branching revealed by immunofluorescence against Iba-1. Relative to the control, LPS reduced the degree of microglial branching (*p* < 0.0001). The flavonoid agathisflavone attenuated this reduction (*p* < 0.0001). However, pretreatment with RU486 reversed the effect of agathisflavone again, reducing the degree of microglial branching (*p* < 0.0001). The relative expression of cluster of differentiation 68 (CD68), a pro-inflammatory microglia/macrophage marker, was also evaluated by immunofluorescence. Relative to the control, LPS increased the expression of CD68 (*p* < 0.0001), but this increase was reduced in the presence of agathisflavone (*p* < 0.0001). However, previous treatment with RU486 attenuated the effect of agathisflavone, increasing CD68 expression (*p* < 0.0001), although it was lower than the LPS-induced levels (Figure 2A,D).

### 2.3. Effects of Agasthisflavone and the GR Antagonist RU486 on LPS-Induced Astrocyte Reactivity

**Figure 3:**
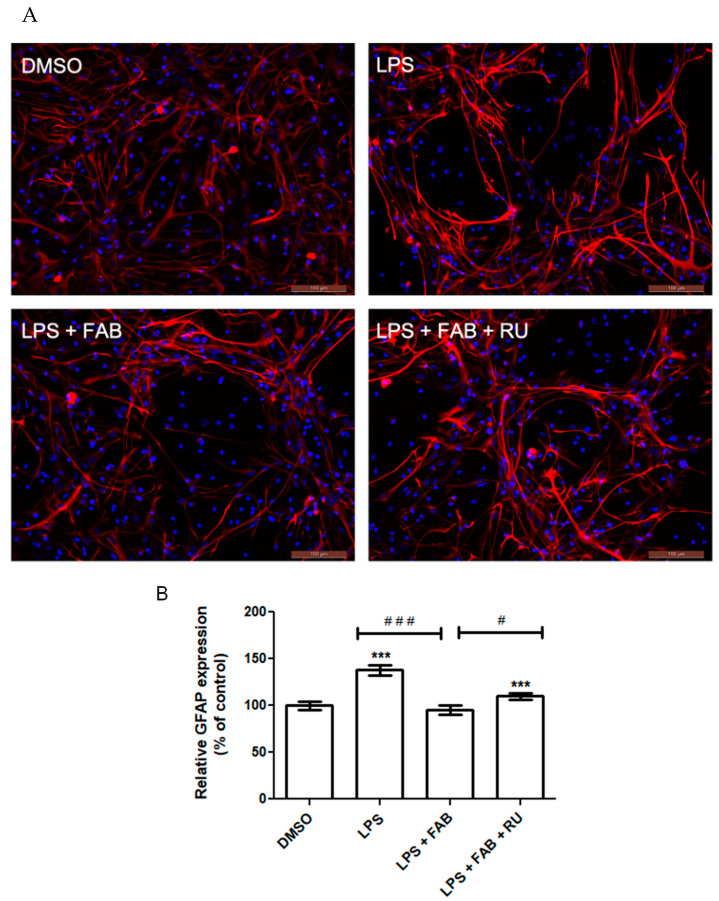
Evaluation of the involvement of GR in agathisflavone effects on LPS-induced astrocyte reactivity. Glial cells were pretreated or not with the GR antagonist RU486 (RU, 1 µM) for 2 h and treated with LPS (1 µg/mL) alone or in association with agathisflavone (FAB, 1 µM) for 24 h. (**A**) Photomicrographs of immunofluorescent staining for glial fibrillary acid protein (GFAP, red) and counterstained with DAPI (blue) for the nuclei. (**B**) Quantification of GFAP relative expression by immunofluorescence in response to treatments; the results are expressed as the mean ± standard deviation relative to the control. The asterisk on the bars indicates a statistical difference relative to control (DMSO 0.001%), which was considered as 100%. The hashes indicate statistical differences between groups; the number of asterisks or hashes corresponds to the significance of the statistical difference, calculated using the Mann–Whitney test (α = 0.05), according to the *p* value: *** *p* < 0.0001; ### *p* < 0.0001; # *p* < 0.05. Scale bars 100 µm.

One of the main features of astrocyte reactivity after inflammatory stimuli is changes in morphology associated with alterations in the expression of its intermediate filament protein, glial fibrillary acidic protein (GFAP) [17]. Hence, we evaluated the effects of agathisflavone in the GFAP expression and morphology by also evaluating the levels of immunofluorescence and measuring the fluorescence intensity. We observed, in the control cultures, astrocytes with typical polygonal to star-like morphologies without any signs of cellular damage, GFAP distributed in the cell body, and short cell processes (Figure 3A). However, treatment with LPS induced changes in the morphology of the astrocytes characteristic of astrocytic reactivity. The cell retracted its body, with a very significant increase in the cellular processes and GFAP expression. On the other hand, the cultures subjected to LPS damage and treated with agathisflavone astrocytes assumed a phenotype like that observed in control cultures. Pretreatment with RU486 reversed the changes in astrocyte morphology observed after LPS and agathisflavone treatment. The GFAP expression was also evaluated by measuring the levels of immunofluorescence and quantified by fluorescence intensity (Figure 3B). In comparison with the control, LPS increased GFAP expression fluorescence intensity (*p* < 0.0001), but this increase was inhibited in the presence of agathisflavone, which restored GFAP expression to control levels. Prior treatment with RU486 attenuated the effect of agathisflavone, increasing GFAP expression, but not to LPS levels (*p* < 0.01).

### 2.4. Effects of Agasthisflavone and the GR Antagonist RU486 on LPS-Induced Cytokines Gene Expression

**Figure 4:**
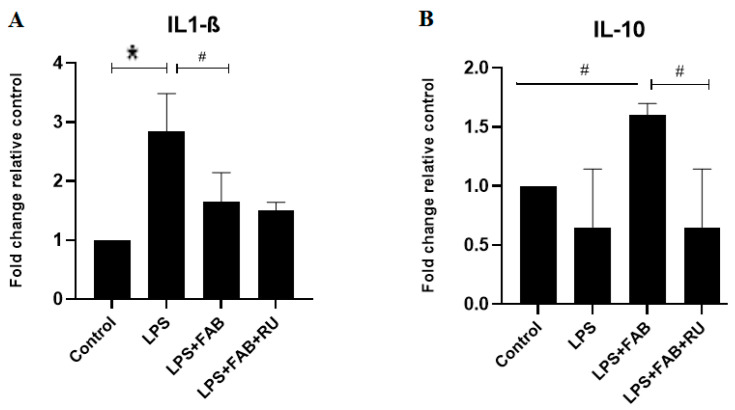
Effects of agathisflavone on inflammatory genes in LPS-treated glial cell cultures. RT-qPCR was performed 24 h after treatment of primary glial cultures with LPS (1 µg/mL), treated or not with agathisflavone (FAB, 1 µM) and pretreated or not with the GR antagonist RU486 (RU, 1 µM) for 2 h. Bar graphs show the relative expression of the genes of: (**A**) interleukin-1β (IL-1β) and (**B**) interleukin-10 (IL-10). Data presented as mean ± SEM fold change relative to the control’s mRNA relative expression. The asterisk (*) on the bars indicates a statistical difference relative to control (DMSO 0.001%). The hashes (#) indicate statistical differences between groups; * *p* < 0.05, # *p* < 0.05.

To better characterize the involvement of GR in the anti-inflammatory effects of agathisflavone on glial cells, we evaluated the mRNA expression of interleukin 1-β (IL1-β), and interleukin 10 (IL-10), key inflammatory markers, by RT-qPCR in cultures exposed to LPS (1 µg/mL) in the presence or absence of the glucocorticoid receptor antagonist RU486 (Figure 4). We observed that LPS significantly increased IL-1β mRNA expression, and agathisflavone treatment decreased LPS-induced IL-1β mRNA expression; this effect was not affected by the GR antagonist (Figure 4A). On the contrary, treatment with LPS decreased IL-10 expression. The treatment with LPS plus agathisflavone increased the mRNA expression of the IL-10 gene, but this increase was reversed by the GR antagonist (Figure 4B).

### 2.5. Analysis of Molecular Docking Interactions of Mifepristone (RU486), Dexamethasone, Agathisflavone, and Apigenin with GR

Three common characteristics are present in all GR structures: a hydrophobic pocket for ligand binding, the conventional three-layer helical sandwich folding, and an active conformation brought on by agonist binding that makes it easier to associate with co-activator proteins [18]. To characterize the interaction of agathisflavone with the glucocorticoid receptor (GR), in silico analyses were performed and compared with the interactions of a known GR antagonist (mifepristone) and a GR agonist (dexamethasone) as well as the agathisflavone monomer apigenin.

**Figure 5:**
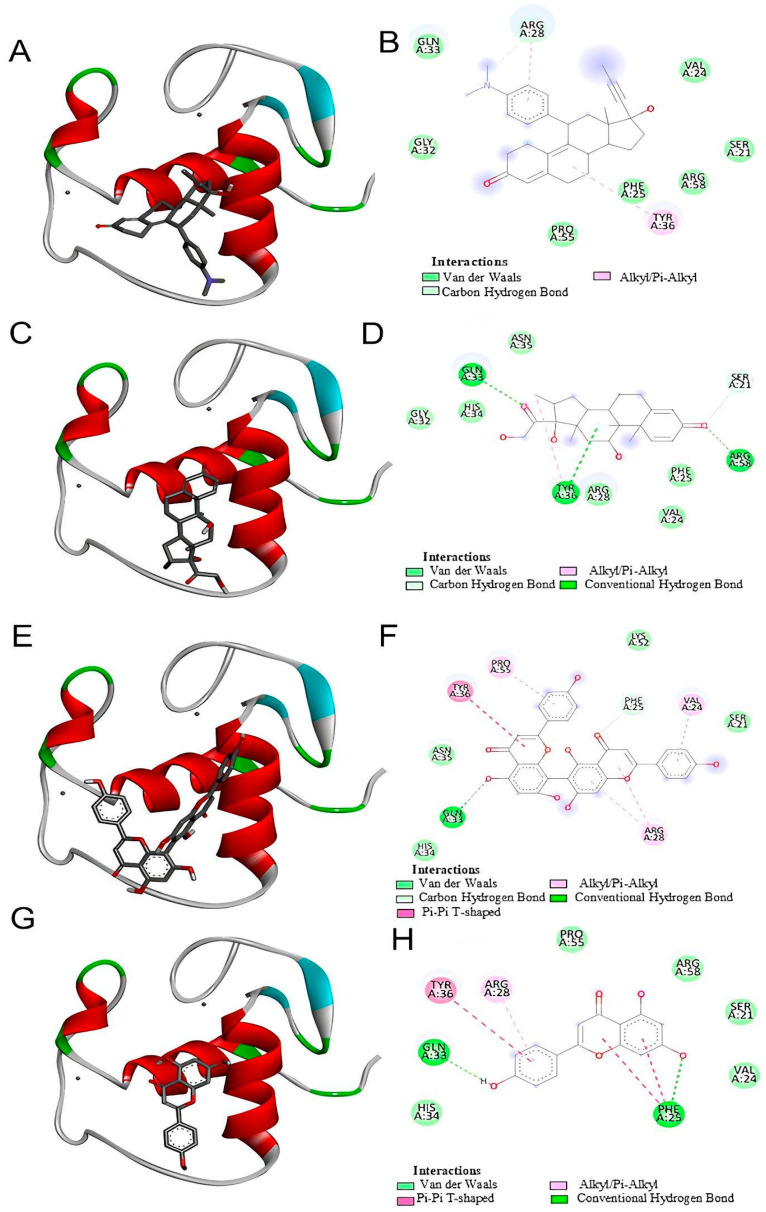
Ligands’ interactions with the GR of *R. norvegicus* (PDB ID 1GDC). Molecular docking of the ligands mifepristone (**A**), dexamethasone (**C**), agathisflavone (**E**), and apigenin (**G**) into a flexible pocket of GR. A 2D diagram demonstrating the amino acids and types of interactions between mifepristone (**B**), dexamethasone (**D**), agathisflavone (**F**), and apigenin (**H**). Dashed lines represent interactions between ligands and amino acids.

Molecular docking showed that all tested ligands bind to the pocket in the glucocorticoid receptor (Figure 5). Agathisflavone, predominantly a non-polar molecule, interacts with the hydrophobic pocket primarily through non-polar interactions. It forms one hydrogen bond with Gln33 along with five Van der Waals bonds, one Pi-Pi T-shaped bond, and three Pi-Alkyl bonds. Gln33 also forms hydrogen bonds with dexamethasone and apigenin, but not with mifepristone. This hydrophobic pocket can adapt its volume to accommodate different ligand sizes, allowing the agathisflavone molecule to fit similarly to its monomer apigenin.

**Figure 6:**
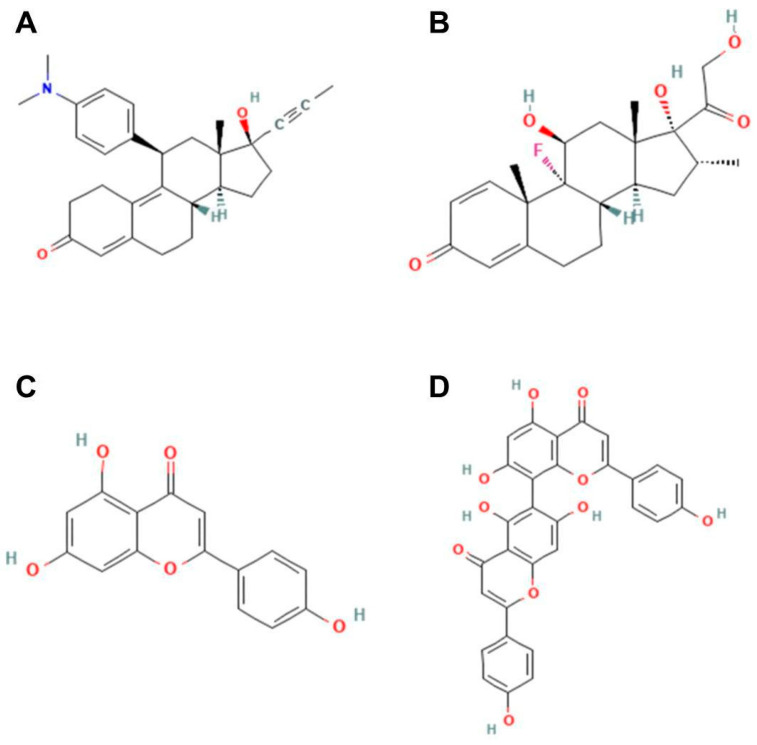
Ligands’ 2D-structure. Mifepristone (**A**), dexamethasone (**B**), apigenin (**C**), and agathisflavone (**D**).

Gibbs free energy is a principle used to analyze the spontaneity of chemical reactions, with more negative values indicating better attraction between molecules. Agathisflavone presents a Gibbs free energy of −8.8 kcal/mol, indicating a stronger interaction compared with mifepristone, dexamethasone, and apigenin (Table 1). The binding energy between agathisflavone and GR suggests a stable interaction, with potential inhibition of the receptor’s activity. The estimated affinity of agathisflavone for the GR allosteric site shows a good correlation with experimental in vitro data. Figure 6 shows the ligands’ 2D-structure of the molecules mifepristone (A), dexamethasone (B), apigenin (C), and agathisflavone (D).

## 3. Discussion

This study investigated whether the glial reactivity induced by agathisflavone is mediated by GR using pharmacological inhibition of the GR receptor with the antagonist RU486 [16] in glial primary cultures. The flavonoid agathisflavone was used at selected concentrations previously described as anti-inflammatory and neuroprotective [12,13]. We chose concentrations of 1 µM agathisflavone and 1 µM RU486 that were not toxic to glial cells after 24 h of treatment (Figure 1).

Unemura and colleagues [16] demonstrated that dexamethasone (0.01, 0.1, and 1 µM) and corticosterone (0.1 and 1 µM) were capable of reducing MTT activity, proliferation, and GR expression in glial cultures enriched with astrocytes after 72 h of treatment. This effect was inhibited by the GR antagonist RU486, suggesting that the proliferation of glial cells could be mediated by GR. The GR knockdown by siRNA also inhibited astrocyte proliferation. Excessive release of glucocorticoids in rats reduced the number of astrocytes in the frontal cortex and the GR expression in the hippocampus and prefrontal cortex, demonstrating that glucocorticoids decrease the number of astrocytes by reducing GR expression [16].

Increased microglial proliferation is a hallmark of pro-inflammatory microglia. Therefore, microglial proliferation is an indicator of pro-inflammatory profiles and microglial reactivity. At the molecular level, inflammatory factors such as LPS and IFN-ϒ induce microglial activation and release pro-inflammatory molecules such as TNFα, IL-1β, and NO, leading to an accentuated microglial population in response to pro-inflammatory stimuli. Functionally, microglial proliferation in response to pro-inflammatory stimuli promotes phagocytosis of cell debris and enhances microglial recruitment by increasing chemokine liberation [19,20].

In the present study, agathisflavone reduced the microglial population and CD68 expression and increased microglial branching during LPS exposure relative to LPS alone. The pretreatment with the GR antagonist, RU486, reversed or attenuated all these agathisflavone effects on microglia. Previous studies have demonstrated that agathisflavone reduces both microglial proliferation and population in response to different damage-inducing inflammations, such as LPS [13].

Koss and colleagues [21] reported that dexamethasone treatment after LPS exposure induces microglia to a primed phenotype, maintaining an increased microglial population during LPS exposure instead of decreasing it. This phenotype is characterized by i. an increased basal expression of activation markers (CD68, MHCII, and other pro-inflammatory molecules), ii. a lower threshold to respond to inflammatory stimuli, and iii. heightened responsiveness to inflammatory stimuli. In the absence of inflammatory stimuli, primed microglia show a decrease in motility and proliferation as well as a reduced ability to shift to an M2 regulatory profile in response to IL-4 and TGF-β. On the other hand, in response to inflammatory stimuli, primed microglia are over-activated, secreting higher levels of pro-inflammatory factors, with higher phagocytic activity and increased proliferation [22]. Microglial morphology, measured through the degree of branching, is one of the indicators of the microglial inflammatory profile. Following LPS-mediated inflammatory stimulation, which makes microglial cells predominantly amoeboid, subsequent treatment with dexamethasone increases the degree of microglial branching, similar to that observed under control culture conditions [21].

The present study demonstrated that agathisflavone increases the degree of microglial branching during exposure to LPS in contrast to exposure to LPS alone, which reduces microglial branching. However, the effect of agathisflavone was significantly attenuated after pretreatment with RU486, a glucocorticoid receptor antagonist. Agathisflavone has been demonstrated to reduce the number of amoeboid microglia, accompanied by a shift to an M2 profile, after 24 h treatment with LPS [13]. Accordingly, the results of this study suggest that agathisflavone depends on GR activity to fully exert the observed anti-inflammatory effect on microglial morphology. 

Macrosialin, or cluster of differentiation 68 (CD68), is a lysosomal protein consistently used as a marker of pro-inflammatory microglia and macrophages. Functionally, its increased expression is suggestive but does not necessarily imply phagocytosis/endocytosis. Phagocytic activity is required to eliminate diseased cells or pathogens during the acute inflammation stage. It also promotes debris removal, which contributes to direct inflammation toward its resolution [23]. In this study, LPS alone increased CD68 expression, while agathisflavone reduced CD68 expression to control levels even in the presence of LPS. Following pretreatment with RU486, the agathisflavone CD68 decrease was attenuated. It has been previously demonstrated that 1 µM agathisflavone reduces CD68 expression, accompanied by an increased CD206 expression after 24 h of LPS exposure in neuron–glia co-culture [13].

In the aforementioned study, Koss and colleagues [21] demonstrated that while dexamethasone restores the microglia-ramified morphology after LPS stimulation, it does not decrease CD68 expression. Considering these results and the findings of Koss and colleagues [21], it is likely that agathisflavone affects microglia inflammatory states via GR and by other pathways. Although its regulatory effect was attenuated by GR antagonists, agathisflavone affected the population of activated microglia expressing CD68. Therefore, it is possible that GR might contribute to agathisflavone activity in conjunction with other transcription factors such as estrogen receptors, already demonstrated to be a target of this flavonoid [12].

Microglia reactivity directly affects astrocytes and vice versa. One of the major methods to characterize astrocyte reactivity is measuring the expression of the intermediate filament protein GFAP, which is the main protein present in the cytoskeleton of astrocytes and is directly implicated in their morphological state and cellular processes (astrocytic feet) [24]. GFAP has long been demonstrated as a reliable astrocytic inflammatory marker due to its consistent and marked increase in expression in response to inflammation and injury [25]. Its expression is modulated by the binding of various transcription factors across the DNA region coding for GFAP.

Among the transcription factors that most markedly increase GFAP expression is NF-κB, which is directly activated by inflammatory stimuli such as LPS binding to its surface receptor, TLR4 [17]. The glucocorticoid receptor is itself a transcription factor, which downregulates NF-κB expression and upregulates NF-κB inhibitors [26]. Therefore, GR activity indirectly suppresses GFAP expression. This study demonstrated that agathisflavone reduces LPS-induced GFAP expression. Following pretreatment with RU486, this reduction was attenuated.

In a study evaluating GFAP expression in the murine hippocampus after LPS injections, it was demonstrated that a 24 h dexamethasone treatment restores GFAP expression to control levels [27]. Although GFAP protein expression increases with inflammatory stimuli and astrocytosis, this does not necessarily imply changes in the number of the astrocyte population [25], as observed in the present study and previously [12].

In the present study, changes in astrocyte and microglia morphologies and states of activation after the potent inflammatory factor LPS were associated with increased expression in mRNA for the inflammatory cytokine IL-1β, which was reversed by agathisflavone alone, and this effect was not affected by the GR antagonist. On the contrary, treatment with LPS plus agathisflavone increased the mRNA expression of the regulatory cytokine IL-10, and this increase was reversed by the GR antagonist. These results suggest that agathisflavone enhances the transcription of IL-10 mRNA via GR activation. Mozo and colleagues [28] observed that different types of glucocorticoids and female steroids such as progesterone and estradiol upregulate IL-10 production in human monocyte cultures. These glucocorticoid effects were abolished by the GR antagonist RU486, suggesting that the glucocorticoid enhancement of IL-10 mRNA is mediated by the binding of the activated GR to the IL-10 promoter gene [28]. Glucocorticoids bind to GR in the cytoplasm, and the complex translocates into the nucleus, where they bind to the glucocorticoid response element (GRE) in the target genes. Glucocorticoids also repress the activity of other transcription factors (e.g., nuclear factor-κB, NF-κB) or initiate gene transcriptions associated with anti-inflammatory effects [16]. De Almeida and colleagues [12] observed that agathisflavone inhibits the expression of the transcription factor NF-κB. This is related to IL-1β downregulation and other anti-inflammatory effects such as the inhibition of microglia activation observed in the present study.

Our in silico analysis showed that agathisflavone binds to a pocket at the glucocorticoid receptor. The agathisflavone interactions with the glucocorticoid receptor were stronger than those of mifepristone, dexamethasone, and apigenin. Three common characteristics are present in all GR structures: a hydrophobic pocket for ligand binding, the conventional three-layer helical sandwich folding, and an active conformation brought on by agonist binding that makes it easier to associate with co-activator proteins [18]. Previous demonstrations showed that agathisflavone relies on estrogen [12] and retinoic acid [11] receptors’ activity to exert its neuroprotective and neurogenic effects, respectively. The binding of agathisflavone to the GR receptor can explain some regulatory effects of this flavonoid on microglial and astrocyte inflammatory profiles that are similar to glucocorticoids.

The interactions between mifepristone (RU486), agathisflavone, and glucocorticoid receptors (GRs) were demonstrated through in silico analysis. However, in our in vitro experiments, this interaction was inferred based on the well-established role of RU486 as a GR antagonist in the literature [29]. Dexamethasone and other corticosteroids are anti-inflammatory GR agonists. Mifepristone is a GR antagonist that blocks the binding of other molecules to the GR. The present study tested whether the anti-inflammatory effects of agathisflavone depend on GR binding through its inhibition by mifepristone. This hypothesis was confirmed. Mifepristone performed its expected function by inhibiting the anti-inflammatory or regulatory effects of agathisflavone on microglia, astrocytes, CD68, and IL-10 gene expression. While agathisflavone was tested for its effects on glial cell cultures, assays to directly confirm its binding to GR could be performed. Therefore, our findings suggest a potential agathisflavone GR-mediated mechanism, but further experimental validation is required to confirm this interaction at the molecular level.

Bioprospecting for new drugs with anti-inflammatory effects that act via GR appears to be essential in medicine. Inflammation is a common phenomenon that affects different systems. Depression, for example, is a neuroinflammatory disease characterized by an excess of pro-inflammatory cytokines and cortisol, due to a phenomenon called glucocorticoid resistance [30]. In depression, increased microglial activity in regions associated with emotional regulation, particularly in the amygdala and prefrontal cortex, has been correlated with increased fear responses and autonomic hyperactivity, features frequently observed in anxiety disorders [31]. Depression increases the risk of greater cognitive decline and of developing dementia and Alzheimer’s disease (AD) by elevating the pro-inflammatory cytokine levels. Antidepressants may reduce the incidence of AD via increased levels of anti-inflammatory cytokines [32]. The search for anti-inflammatory drugs with fewer side effects and known mechanisms of action provides preventive or treatment alternatives for several diseases, including neurodegenerative and neuropsychiatric disorders.

Studies in the literature show the neuroprotective activity of flavonoids and the ability of these compounds to cross the blood–brain barrier [33,34,35]. Moreover, we demonstrated in an in vivo model of spinal cord injury in rats that agathisflavone administered alone at the doses of 10 mg/kg tested protected injured spinal cord tissue and increased the expression of neurotrophins modulating the inflammatory response, which also presumes the capacity to cross CNS barriers [36]. The results of the present study, together with other studies, show the potential use of agathisflavone as an adjuvant in the treatment of neuropathologies in protecting neural cells from inflammatory and other damage, which is relevant considering, for example, the chronicity and lack of effective treatments for neurodegenerative diseases.

## 4. Materials and Methods

### 4.1. Primary Cultures of Glial Cells

Primary cultures enriched with astrocytes were obtained from the cerebral cortex of newborn Wistar rats as previously described [16]; this was carried out according to the Brazilian guidelines for the production, maintenance, and use of animals for teaching and scientific research activities and the Ethics Committee for Animal Use and Experimentation (CEUA protocol No. 6731220818). In brief, the cerebral hemispheres were aseptically collected, and meninges were removed. The tissue was mechanically dissociated, and the cell suspension was gently pushed through a sterile 70 µm Nitex mesh. Cells were centrifuged for 10 min at 1000 rpm and 4 °C and placed in 75 cm^2^ culture flasks in Dulbecco’s Modified Eagle’s Medium (DMEM) (Island Biological Company—Gibco^®^, New York, NY, USA), supplemented with streptomycin 100 μg/mL, 100 IU/mL penicillin G (Island Biological Company—Gibco^®^, New York, NY, USA), 2 mM L-glutamine, 0.011 g/L of pyruvate (Sigma-Aldrich, St. Louis, MO, USA), 10% Fetal Bovine Serum (FBS) (Island Biological Company—Gibco^®^, New York, NY, USA), 10% Horse Serum (HS) (Island Biological Company—Gibco^®^, New York, NY, USA), 3.6 mg/L HEPES (Sigma-Aldrich, St. Louis, MO, USA), and 33 mM glucose and cultured in a humid atmosphere with 5% CO_2_ at 37 °C. After 10 days in vitro, microglial cells were harvested by shaking at 265 rpm at 37 °C for 3 h, the supernatant was removed, and fresh DMEM with 10% FBS was added to the cultures (containing about 90% astrocytes and ~8% microglia) and maintained for an additional 5 days in vitro. For experiments, after 3 washes with PBS, the cells were detached with trypsin solution at 37 °C (Trypsin/EDTA, Sigma-Aldrich, St. Louis, MO, USA), counted in a Neubauer chamber, and plated at a density of 10^5^ cells/cm^2^ on 96-, 24-, or 6-well plates for 3-(4,5-dimethylthiazol-2-yl)-2,5-diphenyltetrazolium bromide (MTT) assay (Thermo Fisher, Waltham, MA, USA, 0.5 mg MTT per 1 mL), immunofluorescence, Western Blot, and RT-qPCR and maintained in culture for 72 h. Cultures with 18 days in vitro were submitted to treatments.

### 4.2. Flavonoid and Treatments

Agathisflavone was purified from the leaves of the Brazilian plant *Poincianella pyramidalis* Tul. as previously described [13], with 99% purity, and diluted in dimethyl sulfoxide (DMSO; Sigma, St. Louis, MO, USA) at 100 mM, forming a stock solution that was protected from light at 4 °C until use.

To assess the cytotoxicity of agathisflavone and mifepristone (RU486, GR antagonist) in the in vitro model used in this study, primary glial cells culture was treated with serum-free DMEM medium containing different concentrations of agathisflavone (0.1 µM, 1 µM, and 10 µM) and RU486 (0.3 µM, 1 µM, and 3 µM) for 24 h. Serum-free DMEM medium containing DMSO 0.003% was used as a control culture.

To elicit an inflammatory response and glial reactivity, glial cells were treated with LPS (1 µg/mL) (Sigma Aldrich, St. Louis, MO, USA). To test if agathisflavone effects were dependent on the glucocorticoid receptor, LPS-stimulated cells were treated for 24 h with agathisflavone (1 µM) either alone or in the presence of RU486 (1 µM). This glucocorticoid receptor antagonist RU486 was added to the culture medium 2 h before the treatments with LPS and agathisflavone. Serum-free DMEM medium with DMSO 0.001% was used as a control.

### 4.3. Cytotoxicity Analysis

To evaluate potential cytotoxicity of substances used in experimental treatments, the 3-(4,5-dimethylthiazol-2-yl)-2,5-diphenyltetrazolium bromide (MTT) test was performed in 96-well plates. Serum-free DMEM medium containing DMSO was used as a control. MTT was diluted in serum-free DMEM to a concentration of 1 g/mL and added to the cells for 2 h at 37 °C in a humidified atmosphere of 5% CO_2_. Then, cells were incubated with sodium dodecyl sulfate buffer (SDS) and dimethylformamide (DMF) (for dissolution and homogeneous distribution of MTT-formazan crystals) overnight at room temperature and protected from light. The absorbance at 595 nm was measured by spectrophotometry using a Varioskan Flash Spectrophotometer (Thermo, Waltham, MA, USA). The cytotoxicity was quantified by measuring the conversion of yellow MTT into purple MTT formazan by dehydrogenases in living cells. Each experimental condition was performed in eight replicate wells in at least three independent experiments, and the results were presented as the percent viability of the control with DMSO, considered as 100% survival.

### 4.4. Immunofluorescence for Astrocytes and Microglia

After experimental treatment, cells were fixed with 4% paraformaldehyde at room temperature for 20 min. Then, the cells were washed with phosphate–saline buffer (PBS) and permeabilized for 15 min with 0.3% Triton X-100 solution diluted in PBS. To block nonspecific binding with antibodies, cells were incubated for 1 h in a 5% bovine serum albumin (BSA) solution diluted in PBS. Then, cells were incubated with primary antibody for 3 h at room temperature in a humidified darkroom. All primary antibody solutions were prepared in PBS containing 1% BSA. For re-blocking, cells were incubated in a 1% BSA–PBS solution for 20 min. Then, cells were incubated with secondary antibody solutions, diluted in PBS for 1 h at room temperature, washed with PBS, and incubated with an intercalating DNA agent (DAPI) for 5 min. After that, cells were washed with PBS and mounted on glass slides with n-propylgallate. Eight images were obtained for each treatment using fluorescence microscopy (Olympus AX70, Olympus, Tokyo, Japan). The following primary antibodies were used at the indicated dilutions: anti-glial fibrillary acid protein (GFAP) (rabbit, 1:500; Z0334, DAKO, Agilent, Santa Clara, CA, USA); anti-calcium-binding protein adapter molecule 1 (Iba-1) (rabbit, 1:500; NBP2-75397, Novus, St. Charles, MO, USA); anti-CD68 (mouse, 1:500; AB53444, Abcam, Cambridge, UK). The following secondary antibodies were used at the indicated dilutions: Alexa Fluor 488 (anti-mouse, 1:1000; A11008, Thermo Fisher, Waltham, MA, USA); Alexa Fluor 594 (anti-rabbit, 1:1000; A110012, Thermo Fisher).

### 4.5. Image Processing of Glial Cells

To evaluate glial reactivity, microglial morphology and relative GFAP expression were evaluated. The morphometric analysis of microglia was performed using the parameters cell area and perimeter, through which we calculated the transformation index (TI). The transformation index is given by the formula TI = (perimeter)^2^/4π (area), which expresses the relationship between perimeter and surface area for each cell. TI expresses how many times the perimeter exceeds the area, and therefore, offers a branching measure. For example, TI = 1 would be obtained from a perfectly round cell. Increasing values of TI indicate acquisition and increased branching [37,38]. Relative GFAP expression levels were measured by fluorescence intensity through densitometric analysis of the immunofluorescence images using the ImageJ software (version 1.5, available at https://imagej.nih.gov/ (accessed on 2 December 2024)).

### 4.6. Quantification of Cytokines Gene Expression by RT-qPCR

To evaluate gene expression after treatment, the culture medium was removed, and then, total RNA was extracted with Trizol^®^ reagent (Invitrogen, Life Technologies, Waltham, MA, USA, 15596026) according to the manufacturer’s specifications. Total RNA concentration and purity were determined by spectrophotometric analysis using KASVI Nano Spectrum (cat# K23-0002, Pinhais, PR, Brazil). To remove DNA contamination, RNA samples were treated with DNase using the Ambion DNA-free kit (cat# AM1906, Life Technologies™). For cDNA synthesis, SuperScript^®^ VILO™MasterMix (cat# MAN0004286, Invitrogen™, Life Technologies) was used with a concentration of 2.5 µg of total RNA in a 20 µL reaction following the manufacturer’s instructions. Quantitative real-time PCR (qPCR) was performed using Taqman^®^ Gene Expression Assays (Applied Biosystems, Foster City, CA, USA) containing a specific Taqman^®^ MGB probe and TaqMan Universal Master Mix II with UNG (cat# 4440038 Invitrogen, Life Technologies^TM^). The assays corresponding to the genes quantified in this study were IL-1β (Rn00580432_m1), IL-10 (Rn01483988_m1), and ARG (Rn00691090_m1). The thermocycling conditions were performed according to the manufacturer’s specifications. The actin beta (ACTB) (Rn00667869_m1) and hypoxanthine phosphoribosyltransferase 1 (HPRT1) (Rn01527840_m1) targets were used as reference genes (endogenous controls) for normalization of gene expression data. Data were analyzed using the 2^−ΔΔCt^ method. The results represent the average of three independent experiments.

### 4.7. Molecular Docking

The 3D structure for the glucocorticoid receptor was retrieved from the RSCB Protein Data Bank https://www.rcsb.org/structure/1GDC (accessed on 25 July 2023) as 1GDC, which corresponds to the refined solution structure of the glucocorticoid receptor DNA-binding domain from *Rattus norvegicus*. The molecule structures for agathisflavone, mifepristone, dexamethasone, and apigenin were downloaded from PubChem https://pubchem.ncbi.nlm.nih.gov (accessed on 25 July 2023). These ligands were downloaded in scf format and converted to pdb using ChimeraX software version 1.8 (University of California, San Francisco, CA, USA). AutoDockTools software version 5.6 (The Scripps Research Institute, La Jolla, CA, USA) was used to prepare proteins and ligands. The water molecules were removed from the receptor and the polar hydrogens, and Kollman charges were added and then saved in pdbqt. For the ligands, Gasteiger charges were added and then saved in pdbqt. After the preparation of the receptor and ligand, the gridbox was performed defining the binding site with the coordinates determined for the insertion of ligands. The docking was performed by the AutoDockVina program using the command prompt programming language. In the end, the 9 best interactions of the ligand with each protein tested were provided, and the one that presented the lowest value of free energy of binding was chosen for analysis. The visualization and analysis of the interactions took place using the BIOVIA Discovery Studio Visualizer 2021 program (Dassault Systèmes, Paris, France). The flavonoid agathisflavone was defined as the ligand and the protein as the receptor. The receptor–ligand interactions were analyzed, and then a 2D diagram was generated that made it possible to visualize the intermolecular bonds and all the amino acid residues present in the interaction between the receptor and the ligands as well as to visualize the interaction in 3D structure.

### 4.8. Statistical Analysis

For both morphometric and densitometric analyses, the statistical tests were performed using the GraphPad Prism software (version 7.0d), and the values of each experimental group were assessed for normality by the D’Agostino and Pearson test and the Shapiro–Wilk test. The experimental groups were compared in pairs using the Student’s *t*-test (if both groups had values in normal distribution) or the Mann–Whitney test (if at least one of the groups had values in non-normal distribution). For RT-qPCR, the results were analyzed by the GraphPad Prism 9.1.1 software (Graphpad, La Jolla, CA, USA). To determine the statistical differences between the groups, an analysis of variance was performed using the One-Way ANOVA test for the parametric data. For nonparametric data, an analysis was performed using Kruskal–Wallis and Dunn’s post-test. In all groups, the results of at least three independent experiments were expressed as the mean ± standard deviation relative to the control DMSO 0.001%, which was considered as 100%. Values of *p* < 0.05 were considered statistically significant.

## 5. Conclusions

In light of the data presented in this study and the aforementioned literature, agathisflavone reduces the inflammation of microglial and astrocyte populations. The ensemble of the results demonstrated that agathisflavone depends on glucocorticoid receptor activity to exert the observed effects on microglial proliferation, morphology, CD68, GFAP, and IL-10 gene expression. The molecular docking analysis showed that agathisflavone binds to a pocket at the glucocorticoid receptor. The agathisflavone interactions with the glucocorticoid receptor were stronger than those of mifepristone, dexamethasone, and apigenin. Altogether, the present study indicates that agathisflavone attenuates microglial and astrocytic pro-inflammatory profiles via GR activity during inflammatory stimulus. The promoted GR function and the consequent increase in the regulatory cytokines’ gene expression contribute to the mechanisms by which agathisflavone exerts its anti-inflammatory effects. The results support the potential use of agathisflavone as a preventive treatment in protecting neural cells from inflammatory damage, which is relevant to neurodegenerative diseases and other neuropathologies. To further elucidate the agathisflavone activity mechanisms, future studies on nuclear transcription factors could investigate whether GR activity is involved in agathisflavone’s effects on glial inflammatory profiles.

## Figures and Tables

**Table 1:** Gibbs free energy and types of interaction of ligands and GR amino acids of *Rattus norvegicus* (PDB ID 1GDC).

Ligand	van der Waals Carbon–Hydrogen Bond	Conventional Hydrogen Bond	Alkyl/Pi-Alkyl	Gibbs Free Energy (kcal/mol)
Mifepristone	Ser21, Val24, Phe25, Gly32, Gln33, Arg58	Not found	Tyr36	−5.8
Dexamethasone	Ser21, Gly22, Val24, Phe25, Arg28, His 34, Asn35	Gln33, Tyr36, Arg58	Not found	−6.0
Agathisflavone	Ser21, Phe25, His34, Asn35, Lys52	Gln33	Arg28, Tyr36, Pro55	−8.8
Apigenin	Ser21, Val24, His34, Pro55, Arg58	Phe25, Gln33	Arg28, Tyr36	−6.2

## Data Availability

Data are contained within the article.
